# Using Service Blueprints to Visualize Pharmacy Innovations

**DOI:** 10.3390/pharmacy7020043

**Published:** 2019-05-08

**Authors:** David A. Holdford

**Affiliations:** Center for Pharmacy Practice Innovation, School of Pharmacy, Virginia Commonwealth University, Richmond, VA 23298, USA; david.holdford@vcu.edu

**Keywords:** serviced marketing, design thinking, medication synchronization, community pharmacists, innovation science, adherence, competitive advantage

## Abstract

**Background**: Applying the principles of service design can help pharmacists manage both the quality and patient perceptions of the services they provide. Service blueprints are a widely used service design tool that are rare in the healthcare literature. They can be used to design new services or revisit the design of established services. This paper describes service blueprints and their uses, and illustrates how to build one using an example. **Methods**: A blueprint is built for appointment-based medication synchronization services to illustrate the tool. **Conclusions**: Service blueprints permit pharmacists to better see and understand service processes. They clarify the process of service delivery and the roles of customers, service providers, and supporting services. They provide a way of depicting complex services in a concise visual way that communicates details at a glance. Pharmacists who utilize service blueprints can improve the consistency and quality of services provided, and they can increase the chance that every interaction with patients sends a positive message about the value of pharmacist services.

## 1. Introduction

Pharmacy is a service profession. Although pharmacy practice revolves around the provision of a tangible product, the value of pharmacists lies in the intangible acts delivered along multiple touchpoints of the service experience. Yet, the public stills sees pharmacists as a dispenser of drugs due to the distinct nature of services [1,2,3,4].

When compared to tangible products, services are much harder to promote due to four distinct attributes they have known as the 4I’s [5]. The first “I” is intangibility, which characterizes services as intangible actions or events. Services are intangible because they cannot be seen, held, or touched. Thus, their quality cannot be measured, tested, or verified in advance of the sale to ensure excellence. The second “I” is inconsistency in that no two service performances are identical—meaning that they vary from person to person, transaction to transaction, and even time to time. Service quality cannot be easily standardized because of the variations among interactions between buyer and seller. The third characteristic is inseparability—referring to the fact that the service provider, customers, and service itself are inseparable because the service is co-created by both the provider and the customer. Each participates in and determines the quality of the service experience. In pharmacy practice, the more engaged the patient, the more likely that better outcomes will occur. The fourth and final characteristic is inventory, which describes how face-to-face services cannot be put into inventory or on a shelf for later use. With online and mobile services, this characteristic is less applicable because technology now allows us to store many services electronically. The fourth “I” only applies to person-to-person services that are provided in a real-time, synchronous manner. When added up, the “4I’s” of services present special challenges in marketing the value of pharmacists [5].

## 2. Challenges in Marketing Pharmacy Services

It is a challenge for pharmacists to get customers to appreciate and desire an intangible product they cannot see or touch. The intangible nature of pharmacist services makes it hard for consumers to mentally appreciate what pharmacists do. It is much easier for people to grasp the purpose of a drug than to comprehend the pharmacist services associated with that drug.

Intangible services are hard for consumers to assess. Although some aspects of pharmacy services are easy to evaluate (e.g., fast, friendly, inexpensive), the clinical and technical aspects of the service experience are difficult to evaluate even with extensive experience. For instance, consumers cannot typically tell if a pharmacist omits critical drug-related information during patient counseling, fails to screen for drug interactions, or misses a chance to intervene in avoiding a drug-related problem. Instead, patients tend to rely on variables that they can assess, such as how a pharmacist looks and acts [6].

The variability of pharmacist services also makes them a challenge to provide and assess. Pharmacists and pharmacy technicians are imperfect human beings whose service performances can vary from minute-to-minute with changing service conditions. Each service experience can be affected by a host of factors including the atmospherics of the work environment, the engagement of patients, the characteristics of individual pharmacists and technicians, and the workload. Demand for service varies throughout the day and week; there are times when business is relatively slow and other times when it gets quite hectic. It is a problem to synchronize customers’ demand for services with the availability of pharmacy personnel to serve them. When combined with the fact that most pharmacist services are performed behind the scenes out of view of the customer, patients have an uneven and unclear understanding of the value of what a pharmacist does.

Applying the principles of service design can help pharmacists manage both the quality and patient perceptions of the service provided [7,8]. Service design is the process of planning and organizing the customer service experience using a number of methods and tools derived from disciplines as diverse as ethnography, systems engineering, management science, and informatics. Service design tools and methods typically attempt to portray services visually, using flow diagrams of the customers, front-facing service providers, and supporting individuals and processes. An established service design tool that would benefit pharmacists is the service blueprint [9].

## 3. Service Blueprints

Service blueprints are pictures or maps of service processes intended to help providers and service marketers to design, deliver, and manage new and established service offerings [10]. They depict the service process and the roles of consumers, service providers, and supporting services involved. These visual depictions help improve the delivery of pharmacy services by identifying gaps and potential points of failure in processes. They are able to improve communications between consumers, pharmacists, support staff, and backstage services by mapping out the various points of contact throughout the service process. Service blueprints can visually communicate complex service processes to stakeholders more clearly and efficiently than verbal descriptions of the services.

Although widely used and accepted by service designers in many industries, evidence of the use of service blueprints is lacking in pharmacy. Holdford and Kennedy [9] used service blueprints in describing the dispensing process for a new prescription and for a smoking cessation program. Other than that single instance, no other mention of service blueprints can be found in the pharmacy literature. This paper will describe how to build service blueprints and discuss how they can be used by pharmacists and when.

## 4. Uses of Service Blueprints

Service blueprints can be used whenever there is interest in improving a service experience. It can be used to design new services or revisit the design of established services. A major benefit of developing a service blueprint is that it forces pharmacists to conduct a detailed analysis of each step in the service process. It challenges pharmacists to question the value of each step and clarify the roles of everyone involved. Pharmacists who utilize service blueprints can improve the consistency and quality of services provided, and they can increase the chance that every interaction with patients sends a positive message about the value of pharmacist services.

Blueprints can be used in research to provide explicit details of interventions. A clearly written blueprint can be used in implementation science to allow comparisons at a glance of the exact nature of service innovations in comparison to the status quo. They are also helpful in describing the competitive advantage of one innovation over another, allowing pharmacists to develop service offerings that are sustainable over time [11].

Service blueprints are especially useful in designing complex services that might include multiple people, processes, and channels of communication. The greater the complexity, the increased likelihood of miscommunication, fumbled handoffs between individuals, and lack of accountability between people. A blueprint can clarify problems and coordinate complicated processes.

Finally, blueprints can be used to promote a positive image of pharmacists [7,9]. Because customers often have difficulty evaluating pharmacist services due to the intangible nature of services, they look for tangible clues to quality. Tangible clues are things such as signs, parking, landscaping, cleanliness, advertising, pharmacy layout, and how service personnel are dressed. Service blueprints can be used to identify these visual and physical aspects of services that customers often use as tangible cues of quality. By seeing services from the customer’s perspective, pharmacists can ensure that these tangible cues send the same positive message of professionalism delivered by other touchpoints of the service experience.

## 5. Components of a Service Blueprint

**Figure 1:**
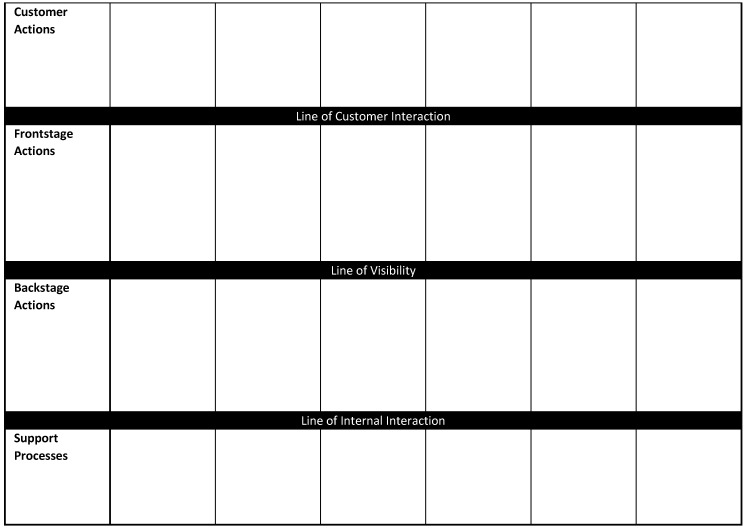
Basic components of a service blueprint.

There are no concrete rules for designing service blueprints allowing for a lot of flexibility in their purpose and use. However, most service blueprints have the following key components [12]: customer actions, “frontstage” contact employee actions, “backstage” contact employee actions, and support processes—as explained below and illustrated in Figure 1.

Customer actions. These are actions performed by the customer when interacting with a service provider. “Customers” can be any recipient of the service including patients, family caregivers, physicians, and nurses, depending on the process. Customer actions associated with a pharmacy visit can be contacting the pharmacy website prior to the visit, driving to the pharmacy, dropping off a prescription, providing information to the pharmacist, waiting, receiving the prescription along with counseling, and leaving the pharmacy. Each customer action is a touchpoint or moment-of-truth where customers judge the quality of pharmacy services and make decisions about whether to continue visiting the pharmacy in the future. The number of customer actions and the level of detail about each depends on the depth of the analysis desired.

Frontstage actions. These are the service actions that are directly visible to the customer. They can be actions between the customer and service personnel or with technology, such as a website or mobile app. Frontstage actions associated with a pharmacy visit could include interfacing with the pharmacy website prior to the visit, talking to pharmacy personnel throughout the steps of the dispensing process, and a follow-up phone call to help resolve any concerns the patient has about their medications. Frontstage actions do not always match every customer action. Some customer actions, like waiting for a prescription to be filled, are things that the customer does alone.

Backstage actions. These are any actions done for the customer that are not visible. Backstage actions in a pharmacy might include professional decisions made by pharmacists (e.g., checking the patient profile for drug allergies, interactions, and duplicate medications), consultations with physicians about therapy, resolving insurance claims, and remote filling of prescriptions.

Support processes. Support processes are any actions that support frontstage and backstage actions in service delivery. For pharmacists, these might include computer support services, billing, prescription claims adjudication, web design, and inventory control.

In service blueprints, key components of the blueprint are separated by three horizontal lines: the line of interaction, the line of visibility, and the line of internal interaction (Figure 1). The line of interaction is where customers and providers interact. Service encounters occur wherever a vertical line crosses the line of interaction. The line of visibility separates frontstage contact employee actions and backstage employee actions. Actions below this line are invisible to the customer. This line is critical because patients’ image of a pharmacist is determined to a great extent by what they see the pharmacist do. If the patient’s primary view is of the pharmacist counting and pouring, then the image of pharmacists will be consistent with that view. The line of internal interaction separates frontline employees from supporting individuals. Any action that crosses this line indicates a process that supports the frontline employee.

### Optional Components of Service Blueprints

Additional components can be added to service blueprints depending on the purpose and goals of completing one. These components include physical evidence, arrows, and time.

Physical evidence. This describes the tangible features of each step in the service process that might influence a customer’s perception of the service experience and service firm. Physical evidence includes the dress of service employees, the signage in the pharmacy, location, advertising, website design, delivery vehicles, and pharmacy layout. These and other forms of tangible evidence set expectations of service and influence evaluation of service quality. Clarifying the physical evidence of services can help ensure a consistent message across all elements of the service experience.

Arrows. Arrows are used to visualize relationships between key components of the blueprints. Arrows are used to indicate dependent relationships between customer and service provider actions. Single arrows indicate linear one-way relationships and double arrows signify collaborative two-way relationships.

Time. Time can be added to service blueprints to map out the amount of time needed for the total service experience or elements of it. This can be useful in estimating personnel costs in providing services and estimating the cost effectiveness of each step in the process.

## 6. Building a Blueprint

Building a service blueprint starts by developing an intimate understanding of the customers’ service experience as well as a clear delineation of all of the people, processes, and systems involved. Whether the pharmacy service experience is face-to-face, online, via an app, over the phone, or via multiple service channels, a service blueprint can be used to map out the process.

Service blueprints can be developed using a provider-centered or a customer-centered perspective. Provider-centered perspectives start by mapping out the details of the service delivery process and then seeing where customers interact with this process. Provider-centered perspectives might be taken when analyzing an established service for gaps, redundancies, and other inefficiencies in delivery. A customer-centered approach starts by mapping out the service experience from the customer’s perspective. It begins by outlining the points of contact between customers and the service—customer experiences with the pharmacy parking lot, the pharmacy building itself, signage, website, phone calls, printed bag, self-service machines, and contact with frontline employees like clerks, technicians, and pharmacists. Each time a customer interacts with a touchpoint, they have a service encounter that is mapped on the blueprint. The following description of building a service blueprint will use a customer-centered perspective.

**Figure 2:**
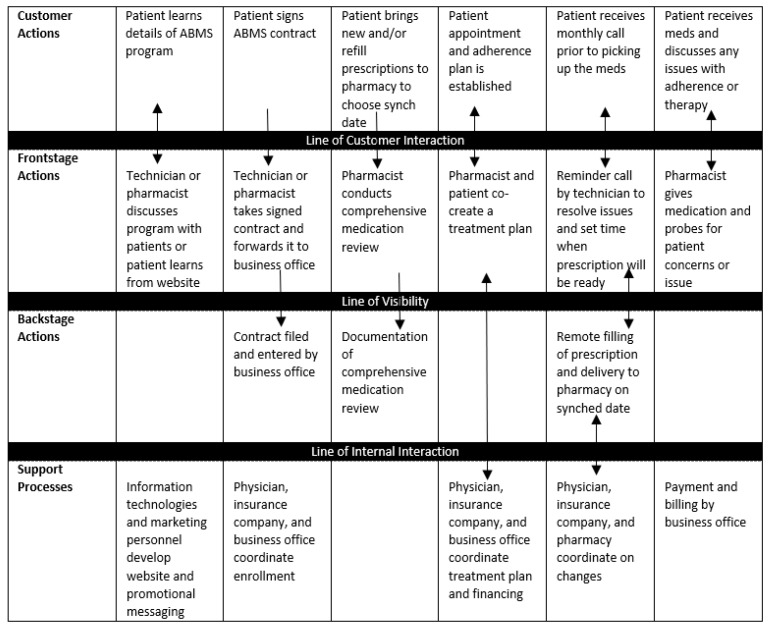
Service blueprint of an appointment-based medication synchronization (ABMS) program.

The process of building a customer-centered service blueprint commences by asking what the customer hopes to achieve by interacting with a pharmacy service. The blueprint will vary depending on whether the visit is to fill a prescription, ask advice, enroll in a smoking cessation program, seek a nonprescription medication, or some other reason. Another question to be asked is the breadth and level of detail desired in mapping the service. An entire service process (e.g., dispensing) can be mapped, or the focus can be on a specific component of the process (e.g., patient counseling). Once these decisions are made, the blueprint is built through the following steps [12], which will be illustrated using the service blueprint illustrating an appointment-based medication synchronization program shown in Figure 2 [13].

1. Draw a template similar to the one in Figure 1 delineating the components of the service blueprint including lines of interaction and any optional components like physical evidence and time. Note that arrows can only be added as the actions are added.
2. Map the customer’s journey through the service experience. From the customer’s perspective, chart each action and choice made by the customer and place them sequentially in the customer actions of the blueprint template. For the appointment-based medication synchronization program in Figure 2, customer actions are patient learns details of the program, signs a contract, brings in prescriptions to the pharmacy to choose synch date, participates in establishing an appointment and adherence plan, receives a monthly call prior to picking up the medications, and receives the medications and discusses any issues with adherence or therapy.
3. Map frontstage actions. List all of the visible points of contact between the customer and specific service personnel, technologies (e.g., website), or processes. They should generally match the temporal sequence of the customer’s journey throughout the service process although there may not always be a matching frontstage action with each customer action. For the program in Figure 2, frontstage actions are a discussion of the program with patients, having the patient sign the contract, the pharmacist conducting a comprehensive medication review, the cocreation of a treatment plan, a reminder call, and the interaction with the patient when they visit the pharmacy.
4. Map backstage actions. List all of the service actions which are invisible to customers. In many cases, these are actions in support of frontstage actions but sometimes they involve interactions with other healthcare professionals, healthcare insurers, and other stakeholders.
5. Map internal support activities. Support activities may serve either frontstage or backstage actions. In Figure 2, internal support activities serve both frontstage and backstage actions and consist of support from information technology, marketing, website design, and the business office.
6. Add additional details as needed including physical evidence, arrows, and time. The blueprint in Figure 2 has single arrows to illustrate one-way relationships (e.g., patient signs the ABMS contract and gives it to a technician or pharmacist). Double arrows illustrate two-way relationships where both customer and provider collaborate in the task (e.g., establishing a patient appointment and adherence plan). Note that some arrows may cross more than one line of interaction.

## 7. Managerial Uses of Service Blueprints

The process of completing service blueprints requires pharmacists to clarify each step of the service experience. This process often leads to obvious opportunities for improving service delivery. Blueprints might suggest a need for minor improvements or a complete overhaul of the service system. The following is a list of actions that might be taken in response to an analysis of a service blueprint:

- Standardize service delivery by ensuring every frontstage, backstage, and support action is consistently provided. Time-and-motion studies and quality improvement cycles can assist in this standardization.
- Identify added service steps that might appeal to other customer segments. For instance, some segments might benefit from comprehensive medication reviews in appointment-based medication synchronization while others may not need or want it.
- Incorporate physical evidence into marketing communications plans. Although some pharmacists only see marketing communications in terms of paid advertising, every touchpoint in the service delivery process is a way of communicating a message of quality to patients. Therefore, cues to quality should be identified within the service experience and changed if needed to provide the messaging desired.
- Simplify service delivery. Look at every step of the process and remove any that do not add value.
- Identify those moments of truth that drive customer perceptions of the service process. There may only be a few touchpoints that drive loyalty to a pharmacy. Make certain that delivery at those touchpoints exceeds customer expectations.

## 8. Conclusions

Service blueprints permit pharmacists to better see and understand service processes. It clarifies the process of service delivery and the roles of customers, service providers, and supporting services. It breaks down the service into components and arranges them according to their purpose.

Service blueprints are rare in healthcare although they can be used in implementation research, interprofessional service delivery, business modeling, quality improvement interventions, and outcomes studies. They provide a way of depicting complex services in a concise visual way that communicates details at a glance. Services blueprints also use a customer-centered approach to design in which each customer action is matched to service providers and processes. When combined with physical cues to quality, pharmacists can send a consistent positive message to customers.

Service blueprints force a careful analysis of each step in the service process and help communicate that information to people such as the frontline employees who help determine its success. When everyone in the service process engages in the development of blueprints, they can understand each step in the process, probe for difficulties, and identify problem areas.

Finally, blueprints can facilitate the analysis of cost–benefit tradeoffs in providing services. They provide a template for quantifying the value of each patient contact, customer touchpoint, and support process. In some cases, some frontstage and backstage actions may be added if they add value to the service experience. In other cases, low value activities may be deleted without any impact on perceived value. Service blueprints allow pharmacists to be more strategic in the design and delivery of their services.
